# A Bayesian generalized random regression model for estimating heritability using overdispersed count data

**DOI:** 10.1186/s12711-015-0125-5

**Published:** 2015-06-20

**Authors:** Colette Mair, Michael Stear, Paul Johnson, Matthew Denwood, Joaquin Prada Jimenez de Cisneros, Thorsten Stefan, Louise Matthews

**Affiliations:** Institute of Biodiversity, Animal Health and Comparative Medicine, Bearsden Road, Glasgow, G611QH UK; School of Veterinary Medicine, Bearsden Road, Glasgow, G61 1QH UK; The Boyd Orr Centre for Population and Ecosystem Health, University of Glasgow, Glasgow, G12 8QQ UK

## Abstract

**Background:**

Faecal egg counts are a common indicator of nematode infection and since it is a heritable trait, it provides a marker for selective breeding. However, since resistance to disease changes as the adaptive immune system develops, quantifying temporal changes in heritability could help improve selective breeding programs. Faecal egg counts can be extremely skewed and difficult to handle statistically. Therefore, previous heritability analyses have log transformed faecal egg counts to estimate heritability on a latent scale. However, such transformations may not always be appropriate. In addition, analyses of faecal egg counts have typically used univariate rather than multivariate analyses such as random regression that are appropriate when traits are correlated. We present a method for estimating the heritability of untransformed faecal egg counts over the grazing season using random regression.

**Results:**

Replicating standard univariate analyses, we showed the dependence of heritability estimates on choice of transformation. Then, using a multitrait model, we exposed temporal correlations, highlighting the need for a random regression approach. Since random regression can sometimes involve the estimation of more parameters than observations or result in computationally intractable problems, we chose to investigate reduced rank random regression. Using standard software (WOMBAT), we discuss the estimation of variance components for log transformed data using both full and reduced rank analyses. Then, we modelled the untransformed data assuming it to be negative binomially distributed and used Metropolis Hastings to fit a generalized reduced rank random regression model with an additive genetic, permanent environmental and maternal effect. These three variance components explained more than 80 % of the total phenotypic variation, whereas the variance components for the log transformed data accounted for considerably less. The heritability, on a link scale, increased from around 0.25 at the beginning of the grazing season to around 0.4 at the end.

**Conclusions:**

Random regressions are a useful tool for quantifying sources of variation across time. Our MCMC (Markov chain Monte Carlo) algorithm provides a flexible approach to fitting random regression models to non-normal data. Here we applied the algorithm to negative binomially distributed faecal egg count data, but this method is readily applicable to other types of overdispersed data.

## Background

Faecal egg count is a commonly used indicator of susceptibility to gastrointestinal nematode infection and provides a marker for selective breeding programs. Infection has traditionally been controlled with anthelmintic drugs, but resistance to these drugs has directed attention towards selective breeding as a sustainable and viable alternative [1]. Selective breeding programs rely on estimates of the animals’ breeding values, which represent the sum of the additive effect of the genes received from both parents [2]. Thus, designing an effective selective breeding scheme requires accurate assessment of the heritability.

The analysis of faecal egg count data is challenging for two reasons. First, the data are overdispersed, which has led previous studies to use transformed faecal egg counts [3, 4]. Transformations of faecal egg count data (commonly a log transformation) can result in bimodal data [5] and therefore may not be appropriate [6, 7]. However, any transformation can be avoided by modelling the raw faecal egg counts as a negative binomial distribution [3]. The second challenge is that, as the adaptive immune response develops, the sources of variation and the heritability of faecal egg counts are expected to change over time, which suggests that a multivariate approach may be appropriate. The goal of this paper was to estimate the change in heritability of faecal egg count over the grazing season.

Random regression models are commonly used to model changes in quantitative traits measured over a continuous scale such as time or age [8]. In particular, they can be used to estimate changes in genetic and environmental variance components as continuous functions over a time frame by specifying time-dependent functions *ϕ*_1_,…,*ϕ*_*k*_ [9]. However, these models can involve the estimation of a large number of parameters that may exceed the number of observations and become computationally intractable, which prompts the use of reduced rank random regression [8]. By estimating each covariance matrix using relatively few principal components, or eigenfunctions, the number of parameters to be estimated can be significantly reduced [8]. Then, the reduced rank random regression models estimate continuous covariance functions using a small number of the largest eigenvalues [10].

Random regression models have been widely used to estimate genetic parameters of repeated measurements over time [11] and, previously, Bayesian methods have been used to capture the skewed distribution of faecal egg count data [12, 13], but because of the methodological challenges, to date, these two approaches have not been combined.

Software is available to fit random regression models, such as WOMBAT [14], ASReml [15] and RRGIBBS [16]. Each program can be used to estimate variance components. WOMBAT fits linear mixed models, of full or reduced rank, through restricted maximum likelihood (REML). ASReml can be used to fit generalised linear mixed models and RRGIBBS fits random regression models using a Gibbs sampler. However, none of these packages can conduct random regression analyses of generalised mixed models that are also reduced rank. For these reasons, we ultimately took a Bayesian approach by fitting a multivariate negative binomial reduced rank random regression model using a Metropolis Hastings algorithm implemented in R [17].

This paper deals with heritability estimates on two scales. Using faecal egg count data collected from five consecutive cohorts of 200 Scottish Blackface lambs, our overall goal was to provide an alternative method to estimate the heritability of faecal egg count on the link scale and identify the short-comings of estimating heritability on a latent scale by giving specific attention to log transformations [7, 18, 19].

The specific steps in this work were to: (1) demonstrate, using our data, the short-comings of the approaches that are commonly used to handle faecal egg count data, in particular, the use of univariate analyses when the data exhibit correlations that are significantly different from zero between variance components over time, and the use of a log transformation when the data follow a negative binomial distribution; (2) we used standard software (WOMBAT) to demonstrate and compare full and reduced rank analyses of the log transformed data; (3) we applied a reduced rank random regression approach, which assumes that the data are negative binomially distributed. Using residual diagnostics, we showed that the random regression model using the untransformed data provided a better model than the random regression based on the log transformed data. Heritability estimates from the random regression model using the untransformed data were similar to those from the univariate analyses in the later months; however, in the early months, the random regression model explained a much greater proportion of the variance and resulted in substantially higher values of the heritability.

## Methods

### Data source

Data were collected over consecutive years from five cohorts of 200 straight-bred Scottish Blackface lambs between 1992 and 1996 [4]. Within each year, lambs were born over a two-week period and monthly faecal egg counts were estimated using standard procedures between May and October with additional post-mortem samples. As is common practice on this farm, immediately after collection of the faecal samples each month, the lambs were given the recommended dose of anthelmintic.

Since eggs are counted in a $\frac {1}{50}$th of a gram of faeces, each egg counted represents 50 eggs per gram [20]. Following standard practice, we use the term faecal egg count (FEC) for eggs per gram of faeces, and subsequently use the term raw egg count to refer to the distribution of counts per $\frac {1}{50}$th gram.

Seven categories of adult nematodes were detected with variable prevalences across the five years. *Teladorsagia circumcincta* was identified for nearly all the lambs analysed [21]. Across the five years, 36 rams and 485 dams were examined.

The data are unbalanced with varying degrees of missingness between months and as much as two-thirds of the data missing in May (Table 1). Post-mortem counts were not measured in the last year of the study and most of the data were collected from male lambs. Due to the unbalanced nature of the data and level of missingness, the first and last time points were removed from all multivariate analyses in this paper.

**Table 1:** Number of records and percentage of missing records for each month and post-mortem counts (PM)

Month	Number of records	% Missing
May	343	66 %
June	503	50 %
July	859	14 %
Aug	881	12 %
Sep	913	9 %
Oct	713	29 %
PM	503	50 %

### Modelling the data

Quantitative traits are often assumed to be normally distributed [22, 23]. Consequently, it has become common practice to fit a normal distribution to log transformed faecal egg counts (FEC) of the form log(FEC+*c*) for some constant *c* [4, 24] or to Box-Cox transform faecal egg counts [25]. However, such transformations can result in bimodal data [5].

Transformation of faecal egg count data may not be necessary [6, 7, 26] since such count data often follow a negative binomial distribution [3]. The negative binomial distribution is parameterised by the arithmetic mean *μ* and a positive exponent *r* [27] and has also been shown to be a good fit to the distribution of faecal egg counts for a wide variety of parasites [5, 28]. In this parameterisation, the variance of the distribution is $\mu (1+\frac {\mu }{r})$ and thus approaches a Poisson distribution as the dispersion parameter *r* increases and so smaller values reflect more dispersion.

We began our analyses of these data by fitting a series of univariate nested half-sib design mixed models to log transformed faecal egg counts and untransformed raw faecal egg counts. We then used a multitrait animal model to quantify temporal correlations, which prompted us to use a random regression model. We used standard software (WOMBAT) to implement full and reduced rank analyses of the log transformed data and finally applied our negative binomial reduced rank random regression model.

### Univariate analysis

In previous studies, heritabilities of log(FEC+1) and log(FEC+25) transformations of these data were estimated by treating each month independently [4, 24]. We examined the use of log transformed faecal egg count data by comparing univariate heritability estimates of log(FEC+*c*) for a range of values of *c* over the seven-month period. We assumed that the log transformed data were normally distributed. For each model, we fitted year of birth and sex as fixed effects. The nlme package in R [29] was used to estimate heritabilities on the latent scale using nested half-sib design mixed models. This model takes the form:

$$\begin{array}{@{}rcl@{}} Y_{i_{j_{k}}}=\mathbf{X_{i}}\pmb{\uptheta}+ S_{k} + D_{j_{k}} + \epsilon_{i_{j_{k}}}, \end{array} $$

where $Y_{i_{j_{k}}}$ is an observation from the *i*-th lamb, *S*_*k*_ is the random effect of the *k*-th sire and $D_{j_{k}}$ is the random effect of the *j*-th dam within the *k*-th sire. Coefficients of fixed effect are denoted by *θ*, *X*_*i*_ is the corresponding design matrix and $\epsilon _{i_{j_{k}}}$ is the residual variance.

We used similar univariate nested half-sib design mixed models to estimate month by month heritabilities for the raw egg count data. The residual variation in these models was estimated using the method described by Tempelman et al. [30]. The R package glmmadmb [31] was used to fit negative binomial mixed effects models.

The MCMCglmm (MCMC generalised linear mixed models) package [32] was used to fit transformed data (log(FEC+1)) to a multitrait animal model to estimate correlations between June and October. Using this method, we estimated pairwise phenotypic, maternal and genetic correlations between the five months simultaneously. Due to the large number of parameters estimated, we compared these results to those that were obtained using a series of bivariate animal models as an informal check of convergence [33]. Any major deviations between the two sets of models would indicate that the chain had not fully converged.

Each model was run for 1 000 000 iterations with a burn-in period of 50 000 iterations. Inverse-Wishart priors were used for each covariance matrix. We constructed these priors such that faecal egg counts accross months were *a priori* independent with the total variance being spread evenly across months [32].

### Multivariate analysis

A log(FEC+1) transformation was used for the multivariate analyses of the transformed faecal egg count. This transformation was chosen for consistency with previous multivariate analyses of these data [4].

Random regression models are mixed effect models with individual functions of continuous covariates fitted as random effects and are commonly fitted by specifying time-dependent basis functions *ϕ*_1_(*t*),…,*ϕ*_*K*_(*t*) [8]. In this paper, we aimed at modelling individual changes in additive genetic, maternal and permanent environmental effects over time. Time refers to month and we assumed that all fixed effects remained constant across months. Sex and year of birth were included as fixed effects in all models.

For lamb *i* with trait values *Y*_*i*_={*Y*_*i*1_,…,*Y*_*iT*_} (where T denotes the number of time points), a random regression model takes the form:

$$\begin{array}{@{}rcl@{}} \mathbf{Y_{i}}&=&\mathbf{X_{i}}\pmb{\uptheta} + \pmb{\Phi}\pmb{\upalpha'_{i}} + \pmb{\Phi}\pmb{\upbeta'_{i}} + \pmb{\Phi}\pmb{\upgamma'_{i}} + \pmb{\upepsilon_{i}}.  \end{array} $$

The matrix *Φ* contains the set of basis functions evaluated across the time frame. The vector *θ* contains fixed effect regression coefficients and *X*_*i*_ is the corresponding data matrix for lamb *i*. Vectors *αi*′, *βi*′ and *γi*′ are individual regression coefficients relating to the additive genetic, permanent environmental and maternal random effects. Note that the number of basis functions can differ between random effects. Addition residual variance is denoted by vector *ε*_*i*_={*ε*_*i*1_,…,*ε*_*iT*_}.

Since any covariance matrix *Σ* can be decomposed as *Σ*=E*λ*E^*T*^, where *λ* is a diagonal matrix of ordered eigenvalues and E an orthonormal matrix of eigenvectors, the matrix can be approximated by setting the smallest eigenvalues (in the matrix *λ*) to zero. Using this decomposition, the random regression model can be written as:

$$\begin{array}{@{}rcl@{}} \mathbf{Y_{i}}&=&\mathbf{X_{i}}\pmb{\uptheta} + \pmb{\Phi} (\pmb{\mathrm{E}_{\alpha}\mathrm{E}_{\alpha}}^{T})\pmb{\upalpha'_{i}} + \pmb{\Phi} (\pmb{\mathrm{E}_{\beta}\mathrm{E}_{\beta}}^{T})\pmb{\upbeta'_{i}}\\ &&+ \pmb{\Phi} (\pmb{\mathrm{E}_{\gamma}\mathrm{E}_{\gamma}}^{T})\pmb{\upgamma'_{i}} + \pmb{\upepsilon_{i}} \\ &=&\mathbf{X_{i}}\pmb{\uptheta} + \pmb{\Phi \mathrm{E}_{\alpha}}\pmb{\upalpha_{i}} + \pmb{\Phi \mathrm{E}_{\beta}}\pmb{\upbeta_{i}} + \pmb{\Phi \mathrm{E}_{\gamma}}\pmb{\upgamma_{i}} + \pmb{\upepsilon_{i}}, \end{array} $$

with *α*_*i*_=E_*α*_^*T*^*αi*′, *γ*_*i*_=E_*γ*_^*T*^*γi*′ and *β*_*i*_=E_*β*_^*T*^*βi*′. This transformation forms the basis of a reduced random random regression model, however a full description is in Meyer and Kirkpartick [8].

#### Random regression using WOMBAT

The implications of a reduced rank analysis of these data were assessed using full and reduced rank random regression models in WOMBAT [14]. This program assumes normally distributed traits and, thus, we used transformed faecal egg count data (log(FEC+1)).

The number of time-dependent basis functions and the number of eigenfunctions are specified by the user. We used *K* to denote the number of basis functions and *M* to denote the number of eigenfunctions used in any reduced rank analyses [8]. Legendre polynomials were used as basis functions. The first three Legendre polynomials are [34]:

$$\begin{array}{@{}rcl@{}} \phi_{1}(t)&=&\frac{1}{\sqrt{2}},\\ \phi_{2}(t)&=&\sqrt{\frac{3}{2}}t,\\ \phi_{3}(t)&=&\sqrt{\frac{45}{8}}t^{2}+\sqrt{\frac{5}{8}}. \end{array} $$

We began with a second order analysis, using two Legendre polynomials (*K*=2) for each variance component. A first order analysis was not considered since including only the first Legendre polynomial would not allow variance components to be functions of time. In total, we considered two, three and four Legendre polynomials (*K*=2, 3 or 4) in full rank analyses (*M*=*K*) and then considered the same range of Legendre polynomials in reduced rank analyses, each with two eigenfunctions (*M*=2). The results with *K*=2 and *M*=1 are also presented.

#### Negative binomial random regression model

We describe a method to estimate heritability using raw faecal egg count data multivariately using a negative binomial random regression model.

Let *Y*_*i*_=(*Y*_*i*1_,…,*Y*_*iT*_) represent the vector of raw faecal egg counts for lamb *i* (*i*=1,…,*L*) measured at time points 1,…,*T*. *T* denotes the number of observed time points and *L* the number of lambs. Here, *T*=5 and *L*=901. To aid readability, we set *T*=5 in the subsequent formulae.

For lamb *i* at time *t*, the negative binomial model with mean *μ*_*it*_ and dispersion *r*_*t*_ can be parameterised as:

$$\begin{array}{@{}rcl@{}} Y_{it} &\sim& NegBin(p_{it},r_{t})\\ p_{it} &=& \frac{r_{t}}{r_{t}+\mu_{it}}, \end{array} $$

[35]. The dispersion of this negative binomial distribution is controlled by the value of *r*_*t*_ with smaller values producing a higher variance [36]. Using a log link function, we set:



with . The vector *θ* contains the fixed effect regression coefficients corresponding to the overall mean, the sex of the lamb and the year the lamb was born. The matrix *X*_*i*_ is the corresponding data matrix. The matrix *Φ* is of dimension 5×*K*, where *K* is the number of Legendre polynomials functions used and 5 is the number of time points in this study. The *tk*-th entry of the matrix ***Φ*** is *ϕ*_*k*_(*t*).

Three variance components are included: an additive genetic, permanent environmental and a maternal effect. For lamb *i*, $\pmb {\upalpha _{i}}=\{\alpha _{i_{1}}, \ldots, \alpha _{i_{M}}\}\phantom {\dot {i}\!}$ are regression coefficients relating to the additive genetic component, $\pmb {\upgamma _{i}}=\{\gamma _{i_{1}}, \ldots, \gamma _{i_{M}}\}\phantom {\dot {i}\!}$ relate to the permanent environmental component and $\pmb {\upbeta _{{dam}_{i}}}=\{\beta _{{dam}_{i_{1}}}, \ldots, \beta _{{dam}_{i_{M}}}\}\phantom {\dot {i}\!}$ relate to the maternal component. The suffix *dam*_*i*_ is used to denote the dam corresponding to lamb *i* and *n*_*d*_ is the total number of dams. *M* is the number of eigenfunctions included in the reduced rank model. Here, we included the same number of polynomial functions and eigenvalues for each of the three variance components. However, the model can easily be extended to include polynomials of different degrees and values of *M*.

Estimated maternal effects can depend on the number of progeny per dam, the number of dams with recorded egg counts and the number of generations of recorded data [37]. In this dataset, there were rarely more than two progeny per dam and for each lamb, only the sire and dam are known. The structure of this dataset is a source of difficulty in distinguishing between maternal additive genetic and maternal environmental effects [4, 37]. Therefore, the maternal effect modelled here may include both genetic and environmental effects. However, the model is easily adapted to specifically model genetic and environment effects [38].

Coefficients *α*={*α*_1_,…,*α*_*L*_} are not independent between lambs due to the pedigree structure. We assumed:

$$\pmb{\upalpha} \sim MVN(\pmb{0},\pmb{\mathrm{A}} \otimes \text{diag}(\lambda_{\alpha_{1}},\ldots,\lambda_{\alpha_{M}})), $$

with A the additive genetic relationship matrix [10, 39].

Coefficients *γ*={*γ*_1_,…,*γ*_*L*_} are assumed independent between lambs:

$$\pmb{\upgamma} \sim MVN(\pmb{0},\pmb{\mathrm{I}_{L}} \otimes \text{diag}(\lambda_{\gamma_{1}},\ldots,\lambda_{\gamma_{M}})), $$

and $\pmb {\upbeta }=\{\pmb {\upbeta _{1}},\ldots, \pmb {\upbeta _{n_{d}}} \}\phantom {\dot {i}\!}$ are assumed to be independent between dams:

$$\pmb{\upbeta} \sim MVN(\pmb{0},\pmb{\mathrm{I}_{n_{d}}} \otimes \text{diag}(\lambda_{\beta_{1}},\ldots,\lambda_{\beta_{M}})). $$

Matrices I_*L*_ and $\pmb {\mathrm {I}_{n_{d}}}$ are identity matrices of dimension *L*×*L* and *n*_*d*_×*n*_*d*_ respectively.

The values $\lambda _{\alpha _{1}}, \ldots, \lambda _{\alpha _{M}}$ are the *M* largest eigenvalues of the additive genetic covariance matrix, and the matrix $\pmb {\mathrm {E}_{\alpha _{M}}}$ contains the corresponding eigenvectors. Similarly, the values $\lambda _{\gamma _{1}}, \ldots, \lambda _{\gamma _{M}}$ and $\lambda _{\beta _{1}}, \ldots, \lambda _{\beta _{M}}$ are the *M* largest eigenvalues of the permanent environmental and maternal covariance matrices respectively, with the corresponding eigenvectors stored in $\pmb {\mathrm {E}_{\gamma _{M}}}$ and $\pmb {\mathrm {E}_{\beta _{M}}}$.

Legendre polynomials are orthogonal [40] and subsequently the matrices $\pmb {\mathrm {E}_{\alpha _{M}}}$, $\pmb {\mathrm {E}_{\gamma _{M}}}$ and $\pmb {\mathrm {E}_{\beta _{M}}}$ and corresponding eigenfunctions are constrained to be orthogonal [41].

All estimates presented in this paper are based on posterior median values from the last 20 000 samples based on 1 000 000 iterations.

#### Specifying prior distributions

Inference was based on Markov chain Monte Carlo simulation and the parameters were updated with a Metropolis-Hastings algorithm. Prior distributions were specified for *θ*, *r*={*r*_1_,…,*r*_5_}, $\{\lambda _{\gamma _{1}}, \ldots, \lambda _{\gamma _{M}}\}$, $\{\lambda _{\beta _{1}}, \ldots, \lambda _{\beta _{M}}\}$, $\{\lambda _{\alpha _{1}}, \ldots, \lambda _{\alpha _{M}}\}$, $\pmb {\mathrm {E}_{\alpha _{M}}}$, $\pmb {\mathrm {E}_{\gamma _{M}}}$ and $\pmb {\mathrm {E}_{\beta _{M}}}$.

We chose to use non-informative priors for regression coefficients relating to fixed effects and incorporated shrinkage priors for variance components [42–44]. Although other priors could naturally be incorporated into this type of model, results presented in this paper are based on the priors described in this section.

Normal priors were used for each element of *θ*, that is:

$$\begin{array}{@{}rcl@{}} \theta_{i} &\sim& N(0, 10^{4}) \hspace{1cm} \text{for}~~ i=1,2,3. \end{array} $$

Using a large variance ensures that the prior distribution is flat over a wide range of values and is centered around zero.

The likelihood function can be penalized to improve estimation of covariance matrices and variance components by shrinking sample eigenvalues towards a given value [42], a property that can be enforced through the prior distribution. We used normal priors for $\log (\hat {\lambda }_{\alpha _{m}})$ [43, 45]:

$$\begin{array}{@{}rcl@{}} \log(\hat{\lambda}_{\alpha_{m}}) &\sim& N(\mu_{\lambda},\sigma_{\lambda}^{2}). \end{array} $$

Similar prior distributions were used for $\log (\hat {\lambda }_{\gamma _{m}})$ and $\log (\hat {\beta }_{\alpha _{m}})$. In this case, we chose to shrink our eigenvalues towards zero with little variation, setting *μ*_*λ*_=0 and *σ*_*λ*_=5. Similarly, variance components can be shrunk towards a value by setting flat gamma priors on corresponding precision parameters [44]. We used gamma priors for each of the five dispersion parameters:

$$\begin{array}{@{}rcl@{}} r_{1}, \ldots, r_{5} &\sim& \Gamma(0.01,0.01). \end{array} $$

Martinez et al. [45] provided a convenient re-parametrisation of a matrix of eigenvectors, E. They defined a matrix *Ω*=(*Ω*_1_,…,*Ω*_*M*_) with $\pmb {\Omega _{m}}=(\Omega _{m_{1}},\ldots,\Omega _{m_{T}})\phantom {\dot {i}\!}$ such that $0 \leq \Omega _{11} \leq \frac {\pi }{2}\phantom {\dot {i}\!}$ and all other entries in $[-\frac {\pi }{2},\frac {\pi }{2}]\phantom {\dot {i}\!}$. The values in the matrix are used to form a matrix of polar coordinates and then transformed to form an orthogonal matrix, E, using a Gram-Schmidt transformation. Following Martinez et al. [45], we set

$$\begin{array}{@{}rcl@{}} p(\pmb{\Omega})&=&\left\{ \begin{array}{ll} \pi^{-4M}, & \hbox{if \(-\pi/2 \leq \Omega \leq \pi/2\) ;} \\ 0, & \hbox{otherwise.} \end{array} \right. \end{array} $$

These transformations were used for $\pmb {\mathrm {E}_{\alpha _{M}}}$, $\pmb {\mathrm {E}_{\gamma _{M}}}$ and $\pmb {\mathrm {E}_{\beta _{M}}}$.

#### Estimating variance components

To estimate variance components as functions of time, we followed the model of Meyer and Kirkpartick [8] and set:

$$\begin{array}{@{}rcl@{}} \hat{\mathcal{G}}(t_{1},t_{2})&=& \sum_{m=1}^{M}{\hat{\lambda}_{\alpha_{m}}\pmb{\hat{\mathrm{E}}_{\alpha_{m}}}^{T}\pmb{\Phi_{t_{1}}}^{T}\pmb{\Phi_{t_{2}}}\pmb{\hat{\mathrm{E}}_{\alpha_{m}}}},\\ \hat{\mathcal{P}}(t_{1},t_{2})&=& \sum_{m=1}^{M}{\hat{\lambda}_{\gamma_{m}}\pmb{\hat{\mathrm{E}}_{\gamma_{m}}}^{T}\pmb{\Phi_{t_{1}}}^{T}\pmb{\Phi_{t_{2}}}\pmb{\hat{\mathrm{E}}_{\gamma_{m}}}} \text{ and,}\\ \hat{\mathcal{M}}(t_{1},t_{2})&=& \sum_{m=1}^{M}{\hat{\lambda}_{\beta_{m}}\pmb{\hat{\mathrm{E}}_{\beta_{m}}}^{T}\pmb{\Phi_{t_{1}}}^{T}\pmb{\Phi_{t_{2}}}\pmb{\hat{\mathrm{E}}_{\beta_{m}}}}, \end{array} $$

for times *t*_1_ and *t*_2_. The function $\hat {\mathcal {G}}$ is the additive genetic covariance function, $\hat {\mathcal {P}}$ is the permanent environmental covariance function and $\hat {\mathcal {M}}$ the maternal covariance function.

Given these functions, the heritability of raw faecal egg counts can be estimated on a continuous time scale. Specifically, at time *t*, the heritability of raw faecal egg counts can be estimated [25] as:

$$\begin{array}{@{}rcl@{}} \hat{h}^{2}_{t} &=& \frac{\hat{\mathcal{G}}(t,t)}{\hat{\mathcal{G}}(t,t)+\hat{\mathcal{P}}(t,t)+\widehat{\mathcal{M}}(t,t)+\hat{\Sigma}^{2}_{e_{t}}},  \end{array} $$

where $\hat {\Sigma }^{2}_{e_{t}}$ is the estimated residual variance at time *t* on a continuous scale. The residual variance in a negative binomial regression can be estimated using the methods described by Tempelman et al. [30]. The negative binomial model is an extension to a Poisson model and there are two sources of residual variance. There is variation in the Poisson sampling and some additional variation due to the overdispersion that is not captured in the Poisson model [7, 19]. Following these methods, at observation time *t*, we approximated the residual variation from the Poisson sampling by $\bar {\lambda _{t}}^{-1}$ and estimated the additional variance as:

$$\begin{array}{@{}rcl@{}} \phi^{(1)}(r_{t}) &=& \frac{\partial^{2}\Gamma(r_{t})}{\partial^{2}r_{t}}.\\ \end{array} $$

We assumed the residual covariance matrix *Σ*^2^ to be a 5×5 diagonal matrix with the *t*-th diagonal entry equal to $\hat {\sigma }^{2}_{e_{t}}=\phi ^{(1)}(r_{t})+\hat {\bar {\lambda _{t}}}^{-1}$. Given the estimated covariance matrix, a continuous residual variance function $\hat {\Sigma }^{2}_{e_{t}}$ was estimated by the methods described by Kirkpatrick et al. [34]. For consistency, we used the same number of Legendre polynomials and eigenfunctions to estimate $\hat {\Sigma }^{2}_{e_{t}}$ as that used to estimate $\hat {\mathcal {G}}$, $\hat {\mathcal {P}}$ and $\hat {\mathcal {M}}$.

The R codes that were used to analyse these data are available on request to the corresponding author.

#### Application to other distributions

It should be noted that this model can be applied to other distributions. In the case of a normally distributed trait, we would assume:



The prior distribution specified for $\sigma ^{2}_{\mu _{t}}$ corresponds to a flat prior that shrinks the residual variances towards zero [44]. Heritability in this case is estimated as:

$$\begin{array}{@{}rcl@{}} \hat{h}^{2}_{t} &=& \frac{\hat{\mathcal{G}}(t,t)}{\hat{\mathcal{G}}(t,t)+\hat{\mathcal{P}}(t,t)+\widehat{\mathcal{M}}(t,t)+\hat{\sigma}^{2}_{\mu_{t}}}.  \end{array} $$

#### Model selection

By fixing the value of *M* (number of eigenfunctions) and increasing the value of *K* (number of Legendre polynomials), we increased the number of polynomials used but fixed the number of components used to estimate each covariance matrix. Therefore, there was little change in the number of parameters estimated between models. However, since a large number of parameters was estimated compared to the number of observations, we compared these models using a bias corrected Akaike information criterion (AIC _*c*_) [46]:

$$\begin{array}{@{}rcl@{}} \text{AIC}_{c}&=&-2\mathcal{L}(y)+2p\bigg(\frac{n}{n-p-1}\bigg), \end{array} $$

where $\mathcal {L}(y)$ is the log likelihood of the data given the estimated parameters, *p* is the number of parameters estimated and *n* the total number of observations.

We used this model selection criterion because a large number of parameters were estimated with respect to the sample size and because, in such a complex model, AIC _*c*_ enforces a higher penalty than AIC, BIC (Bayesian information criterion) or DIC (deviance information criterion).

## Results

**Fig. 1:**
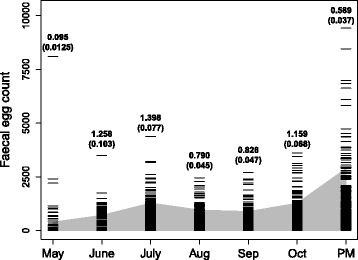
Faecal egg count distributions over time. Faecal egg counts between May and October and at post-mortem counts (PM), black bars. The grey shaded area shows the lower 95 % of the distribution of faecal egg counts over each month. Maximum likelihood estimates of dispersion parameters for the raw faecal egg count data for each month separately and estimated standard errors are given above each bar. Smaller values indicate more dispersed data

**Fig. 2:**
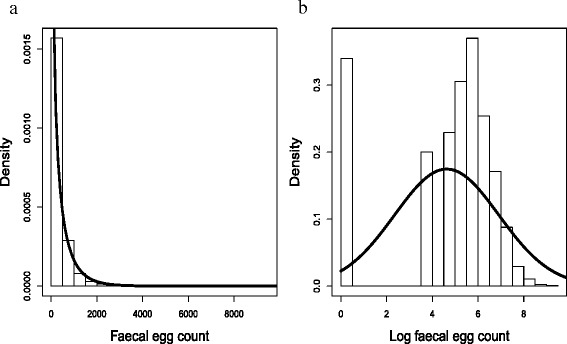
Transformed and raw faecal egg counts. (**a**) Distribution of combined raw faecal egg count data (i.e. the number of eggs counted) over the seven months. The black curve shows the maximum likelihood negative binomial distribution. (**b**) Distribution of log transformed faecal egg count data over the seven months. The transformation used was log(FEC+1). The solid black line shows the maximum likelihood normal distribution

Faecal egg counts (FEC) were quite dispersed (Fig. 1, black bars). Exposing the lower 95th percentile of each distribution (shaded grey area) shows that as the grazing season progresses, the data are more variable and higher levels of dispersion are observed in May, August, September and in the post-mortem counts (Fig. 1, black bars). In particular, raw faecal egg counts are not normally distributed (Fig. 2a) and performing a log transformation i.e. log(FEC+1) produced a mass around zero in our data (Fig. 2b).

Figure 2a shows that the raw faecal egg counts are consistent with a negative binomial distribution (*χ*^2^ goodness of fit test p-value = 0.7276). The log transformed FEC data (Fig. 2b) are significantly different from a normal distribution (Shapiro-Wilk test p-value < 0.001). In addition, log transformed FEC were not normally distributed for *c*=25 or 0.2 (Shapiro-Wilk test p-value < 0.001, in both cases).

Treating each month as a separate trait, we estimated the additive genetic ($\widehat {\text {\pmb {G}}}_{\textit {corr}}$), maternal ($\widehat {\text {\pmb {M}}}_{\textit {corr}}$) and phenotypic (${\widehat {\text {\pmb {Total}}}}_{\textit {corr}}$) correlation matrices as:

$$\begin{array}{@{}rcl@{}} \widehat{\text{\pmb{G}}}_{corr} &=& \left(\begin{array}{ccccc} 1 & & & & \\ -0.53^{*} & 1 & & & \\ -0.52^{*} & 0.56^{*} & 1 & & \\ -0.42^{*} & 0.46^{*} & 0.55^{*} & 1 & \\ -0.17 & 0.32 & 0.49^{*} & 0.51^{*} & 1 \\ \end{array} \right)\\ \\ \\ \widehat{\text{\pmb{M}}}_{corr} &=& \left(\begin{array}{ccccc} 1 & & & & \\ -0.10 & 1 & & & \\ -0.23 & 0.14 & 1 & & \\ -0.08 & 0.03 & 0.20 & 1 & \\ 0.01 & 0.06 & 0.33 & 0.17 & 1 \\ \end{array} \right),\\ \\ \\ {\widehat{\text{\pmb{Total}}}}_{corr}&=& \left(\begin{array}{ccccc} 1 & & & & \\ -0.13^{*} & 1 & & & \\ -0.15^{*} & 0.22^{*} & 1 & & \\ -0.14^{*} & 0.18^{*} & 0.27^{*} & 1 & \\ -0.07 & 0.27^{*} & 0.25^{*} & 0.31^{*} & 1 \\ \end{array} \right). \end{array} $$

An asterisk highlights correlations that differ significantly from zero. We also computed a series of bivariate animal models to estimate pairwise phenotypic, maternal and genetic correlations and found no significant differences between correlations computed in the bivariate models and in the full multitrait model. In addition to running multiple chains, this provided confidence that the chain had converged.

Maternal correlations were not significant. Genetic correlations between July, August, September and October were strong and positive and between June and the remaining four months were strong and negative. Genetic correlations between June and October and July and October were not significant. Phenotypic correlations increased as the season progressed with a strong phenotypic correlation between September and October.

***Fixed effects***

**Fig. 3:**
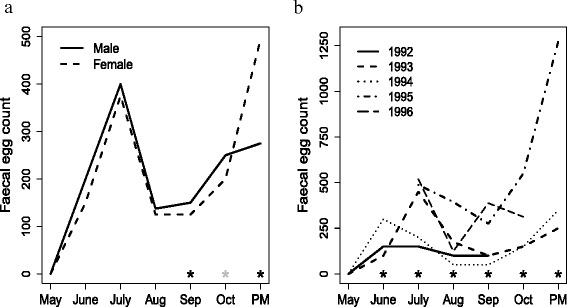
Effects of sex and year of birth. (**a**) Median faecal egg counts for male and female lambs from May to October with additional post-mortem counts (PM). The black asterisk shows significant p-values (<0.05) and the grey asterisk shows a p-value of 0.07. (**b**) Median faecal egg counts between years 1992 to 1996. A black asterisk indicates significantly different counts between the five years

In all analyses, sex and year were significant fixed effects. Considering each month separately, female lambs consistently had lower faecal egg counts than male lambs with the exception of the post-mortem counts. These differences were significant for the last three months (Fig. 3a).

Differences in faecal egg counts across the five years during June to October and in the post-mortem counts were significant (Fig. 3b). Generally, lower faecal egg counts were recorded for years 1992 and 1994 and higher egg counts were recorded for years 1995 and 1996.

***Univariate analysis***

**Fig. 4:**
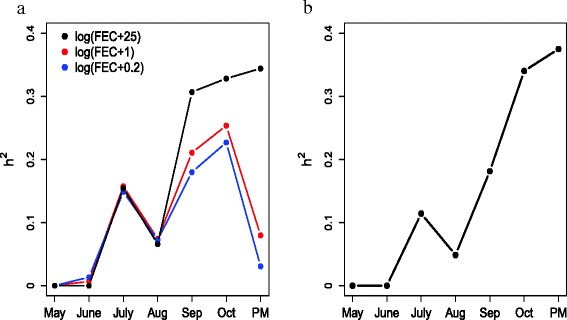
Univariate heritability estimates. (**a**) Heritability estimates of log transformed data. The transformations were of the form log(FEC+*c*) for a range of c values (*c* = 25 black line, *c* = 1 red line, *c* = 0.2 blue line). (**b**) Heritability estimates of raw faecal egg counts

Heritabilities estimated on the latent scale depended on the additional constant used in the log transformation (Fig. 4a). Therefore, heritability on this transformed scale depended on the particular transformation used and thus, did not provide robust heritability estimates. Heritability on the link scale, using a log link function in the negative binomial model of the untransformed raw count data, showed that the heritability of raw faecal egg counts increased univariately from 0 to 0.4 with a dip in August (Fig. 4b).

***Random regression using WOMBAT***

We compared the full rank analyses with *K* = *M* = 2, *K* = *M* = 3 and *K* = *M* = 4, and also compared each reduced rank analysis (with *M* = 1 and *K*=2 or *M*=2 and *K*=3 and 4) with the corresponding full rank analysis. The reduced rank analysis model using four Legendre polynomials (*K* = 4) and two eigenfunctions (*M* = 2) minimised the AICc marginally (Table 2).

**Table 2:** AIC _*c*_ for a range of random regression models with *K* basis functions and *M* eigenfunctions using WOMBAT

*K*	*M*	AIC _*c*_
2	1	7973.300
2	2	7978.478
3	2	7816.062
3	3	7819.068
4	2	7703.478
4	4	7703.922

**Fig. 5:**
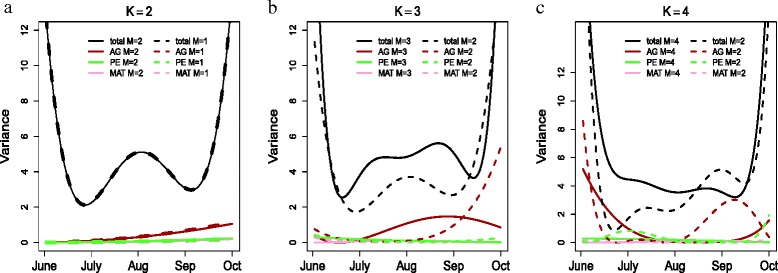
Variance components estimated from log transformed faecal egg counts using WOMBAT. (**a**) Additive genetic (AG, brown), permanent environmental (PE, green) and maternal (MAT, pink) variance components for a full rank analysis with two Legendre polynomials and two eigenfunctions (*K*=*M*=2, solid curves) and for a reduced rank analysis with one eigenfunction (*K*=2, *M*=1 dotted curves). (**b**) Additive genetic (AG, brown), permanent environmental (PE, green) and maternal (MAT, pink) variance components for a full rank analysis with three Legendre polynomials and three eigenfunctions (*K*=*M*=3 solid curves) and for a reduced rank analysis with two eigenfunctions (*K*=3, *M*=2 dotted curves). (**c**) Additive genetic (AG, brown), permanent environmental (PE, green) and maternal (MAT, pink) variance components for a full rank analysis with four Legendre polynomials and four eigenfunctions (*K*=*M*=4 solid curves) and a reduced rank analysis with two eigenfunctions (*K*=4, *M*=2 dotted curves). The transformation used was log(FEC+1). The model with four Legendre polynomials and two eigenfunctions (C, dotted lines) provided the best fit to the data under model selection based on AIC _*c*_

In general, including more Legendre polynomials gave less smooth curves but smaller values of AIC _*c*_ (Table 2). We found little difference in variance components between setting *M*=2 and *M*=1 with *K*=2 (Fig. 5a) and small discrepancies between setting *M* = 2 and *M* = 3 with *K* = 3 (Fig. 5b). However, differences were greater between setting *M*=2 and *M*=4 with *K*=4 (Fig. 5c). Therefore, a reduced rank analysis with higher degree polynomials had a greater effect on variance component estimates.

***Negative binomial random regression model***

The full rank scenarios were computationally infeasible due to the length of time required for convergence. Therefore, we considered reduced rank scenarios that were similar to those presented in the previous section. We used two eigenfunctions (*M* = 2) with two, three and four Legendre polynomials (*K* = 2, 3 and 4). The model using *K* = 3 minimised AIC _*c*_ and thus, gave a better fit compared to *K*=2 or *K*=4 (Table 3).

**Table 3:** AIC _*c*_ for a range of models with *K* basis functions and *M* eigenfunctions using the negative binomial random regression model developed in this paper

*K*	*M*	AIC _*c*_
2	2	-13144.65
3	2	-14453.43
4	2	-14069.68

**Fig. 6:**
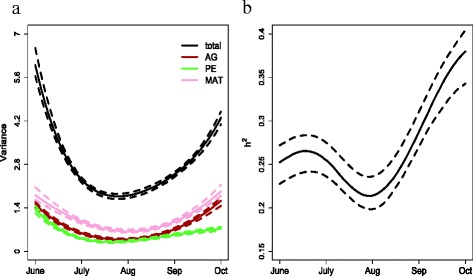
Variance components estimated for raw faecal egg count data using the MCMC method. (**a**) Estimated variance components for the best fitting model which has three Legendre polynomials and two eigenfunctions (*K*=3 and *M*=2). The 95 % credible regions (dotted lines) and median value (solid lines). Black, brown, green and pink curves show total phenotypic variation, additive genetic, permanent environmental and maternal components. (**b**) Estimated heritability

We expected our variance curves to behave similarly to the WOMBAT curves with possible inflated variance estimates at the boundaries of the dataset (Fig. 5). The phenotypic variance increased between July and October (Fig. 6a, black lines). Likewise, the additive genetic component and maternal component showed a gradual rise across months (Fig. 6a, brown and pink lines, respectively). The permanent environmental variance also increased slightly between July and October (Fig. 6, green lines).

A heritability of 0.25 was estimated for June (Fig. 6b), but it may be influenced by inflated boundary estimates. It rose from 0.25 between June and July to 0.4 in October with a dip in August. This dip coincided with a similar pattern found in the univariate analysis (Fig. 4b).

***Residual diagnostics***

**Fig. 7:**
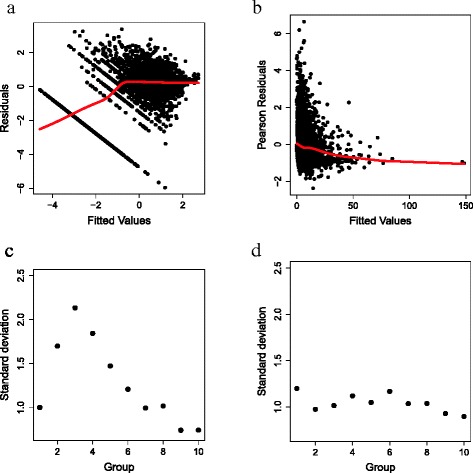
Residual plots. (**a**) Residuals against standardized fitted values from the best fitting normal random regression model in WOMBAT (*K*=4 and *K*=2). (**b**) Pearson’s residuals against fitted values from the best fitting negative binomial random regression model (*K*=3 and *M*=2). In both plots, LOESS lines are indicated by red solid lines. (**c**) Standard deviation of residuals (divided into ten groups) from the best fitting normal random regression model in WOMBAT. (**d**) Standard deviation of residuals (divided into ten groups) from the best fitting negative binomial random regression model

By plotting residuals against the fitted values (Fig. 7), we visually assessed the validity of two key assumptions of the normal log(FEC+1) and the negative binomial linear regression models: linearity and homoscedasticity (constant residual variance). In the negative binomial model, the assumption of homoscedasticity applies only to Pearson residuals, which adjust for the expected mean-variance relationship.

The residuals from the best fitting model of log(FEC+1) (setting *K*=4 and *M*=2 in WOMBAT) showed a strong nonlinear relationship with the fitted values (Fig. 7a, red line) whereas only a very weak trend was observed in the Pearson residuals from the best fitting model of raw faecal egg counts (setting *K*=3 and *M*=2 in the negative binomial random regression model; Fig. 7b, red line).

Homoscedasticity was difficult to assess in the normal model due to the distinct stripe of points on the lower left corner of the plot. Pearson residuals from the negative binomial model appeared to vary more for low fitted values, apparently violating the assumption of homoscedasticity. However, interpretation was also difficult due to the much higher density of points at low fitted values. To informally assess homoscedasticity, we separated the ordered fitted values into ten equally sized groups and calculated the standard deviation of the corresponding residuals within each group. Variation in the standard deviations along the fitted values was substantial for the normal model and was twice as large for mid-level fitted values compared to low and high fitted values (Fig. 7c). By contrast, Pearson residuals from the negative binomial model showed little variation in standard deviation along the fitted values (Fig. 7d).

These observations indicate that the negative binomial random regression model provides a considerably better fit to raw faecal egg count data than the normal random regression of log transformed data.

## Discussion

The overall goal of this work was to present some of the challenges that are inherent in the estimation of heritabilities from faecal egg count data and to demonstrate the use of random regression models to estimate heritability over time on a link scale using untransformed data.

Faecal egg count data are typically overdispersed. Consequently, these data have previously been modelled both univariately and multivariately by transforming faecal egg count data and assuming that they are normally distributed. Our univariate analysis showed that heritability estimates depend on the additional constant used in the log transformation, as was demonstrated in previous studies [47]. The effectiveness of log and Box-Cox transformations decreases as the number of animals with zero egg counts increases [47]. We found that by increasing the additional constant in the log transformation, the heritabilities on the latent scale approached heritabilities on the link scale (Fig. 4). In a separate analysis, we estimated significant phenotypic and genetic correlations between faecal egg counts taken at different times during the season. This suggested that a multivariate analysis such as random regression was more appropriate for these data.

Our paper presents a method to fit a random regression that is based on MCMC. This may be viewed as computationally inefficient compared to REML estimates (for example, as implemented in WOMBAT [14]); however, Bayesian MCMC methods are appealing since they allow for more flexible fitting to complex models which is advantageous in the genetic analysis of non-normal data [18]. Here, we modelled untransformed raw faecal egg counts assuming that they were negative binomially distributed although this method can be easily adapted for fitting longitudinal data following other distributions.

Various studies have compared heritability estimates from sire or animal models based on REML and MCMC methods using real and simulated data [44, 48–50]. Studies on real data have reported that REML estimates are more conservative than the corresponding MCMC estimates [48, 49]. Previous studies showed the importance of prior specification of variance components and adequate mixing in Bayesian analysis [44]. Incorporating more informative priors, for example shrinkage priors, can produce more precise and accurate variance component estimates. However, imposing strict penalties in shrinkage priors inevitably affects parameter estimates. Generally, using more informative priors is beneficial in this type of analysis given the large number of parameters to be estimated. We chose non-informative priors for fixed effect regression coefficients and shrinkage priors for variance components. It is possible to impose stricter penalties [43] and a range of other prior distributions, however, this type of sensitivity analysis was outside the scope of this paper.

Random regressions require fitting polynomial functions, but larger amounts of well-distributed data are required to fit high order polynomials [51]. Consequently, these methods are typically used in systems in which it is straightforward to generate much larger datasets than the one used here [52–54]. To address this issue, and reduce the number of parameters to be estimated, we conducted reduced rank analyses that estimated variance functions by eigen-decomposition of the corresponding covariance matrices of the random regression coefficients.

The reduced rank approach fits a number of polynomial functions (*K*) over time and estimates covariance functions using the largest few eigenfunctions. Analyses of the log transformed faecal egg counts, using the software WOMBAT, showed that decreasing the number of eigenfunctions (*M*) produced more differences compared to the full range analysis (Fig. 5) [55]. For all WOMBAT models considered here, we found possible inflated estimates of variance components at the boundaries of our dataset and consequently, we believe that there are inaccuracies in our reduced rank curves at the boundaries of the data (Figs. 5 and 6). However, this could also be the result of insufficient data to adequately estimate each of the covariance matrices.

Our analyses confirm findings that were obtained with other systems i.e., that heritabilities are not necessarily constant [23] and our results showed that the heritability of faecal egg counts increased as lambs got older, which is expected since exposure of grazing lambs to nematodes helps build immunity to infection. Heritabilities estimated with the MCMC random regression developed here ranged from 0.2 to 0.4 (Fig. 6) and were consistent with those on the latent scale from previous studies [4, 24].

Faecal egg count is a commonly used marker in selective breeding programs for resistance to gastrointestinal nematodes and the heritability of such resistance markers predicts the effectiveness of breeding programs. Our negative binomial model provides estimates of heritability of raw faecal egg counts on a different scale to that used in previous analyses of log transformed data, however, breeding values on different scales tend to be highly correlated [56] and therefore can be interpreted in a similar way to those obtained using the normal animal model.

There is no correct scale on which to measure heritability. Statistical convenience and correspondence with the infinitesimal model in quantitative genetics has often led to calculate heritabilities for faecal egg counts on the latent scale (i.e. following log transformation). However, overdispersed count data such as that obtained here may also arise from multiplicative processes, providing a biological justification for the log link function [18]. We found that the negative binomial random regression model to provided a good fit to raw faecal egg counts whereas residual assumptions of the normal random regression model using log transformed faecal egg counts were not satisfied, which provided evidence in favour of the negative binomial model. Overall, our results agree with previous analyses and demonstrate that raw faecal egg count is a highly heritable trait.

In summary, random regression is a useful tool to analyse variance components from multivariate traits or, in this instance, multiple records of a trait per animal spread over a trajectory. To our knowledge, this is the first time random regression analyses have been used to handle non-normal data on the link scale. We have demonstrated the use of a Bayesian MCMC approach to apply random regression models to negative binomially distributed data. However, the approach taken here can be easily adapted to model data that are consistent with other non-normal distributions.
